# Vagal nerve stimulation protects cardiac injury by attenuating mitochondrial dysfunction in a murine burn injury model

**DOI:** 10.1111/jcmm.12049

**Published:** 2013-04-12

**Authors:** Xiaojiong Lu, Todd Costantini, Nicole E Lopez, Paul L Wolf, Ann-Marie Hageny, James Putnam, Brian Eliceiri, Raul Coimbra

**Affiliations:** Division of Trauma, Surgical Critical Care and Burns, Department of Surgery, University of California San Diego Health SciencesSan Diego, CA, USA

**Keywords:** burn injury, vagal nerve stimulation, cardiac injury, mitochondrial dysfunction, mitochondrial swelling, PI3K/Akt

## Abstract

Mitochondria play a central role in the integration and execution of a wide variety of apoptotic signals. In the present study, we examined the deleterious effects of burn injury on heart tissue. We explored the effects of vagal nerve stimulation (VNS) on cardiac injury in a murine burn injury model, with a focus on the protective effect of VNS on mitochondrial dysfunction in heart tissue. Mice were subjected to a 30% total body surface area, full-thickness steam burn followed by right cervical VNS for 10 min. and compared to burn alone. A separate group of mice were treated with the M_3_-muscarinic acetylcholine receptor (M_3_-AchR) antagonist 4-DAMP or phosphatidylinositol 3 Kinase (PI3K) inhibitor LY294002 prior to burn and VNS. Heart tissue samples were collected at 6 and 24 hrs after injury to measure changes in apoptotic signalling pathways. Burn injury caused significant cardiac pathological changes, cardiomyocyte apoptosis, mitochondrial swelling and decrease in myocardial ATP content at 6 and 24 hrs after injury. These changes were significantly attenuated by VNS. VNS inhibited release of pro-apoptotic protein cytochrome C and apoptosis-inducing factor from mitochondria to cytosol by increasing the expression of Bcl-2, and the phosphorylation level of Bad (pBad^136^) and Akt (pAkt^308^). These protective changes were blocked by 4-DAMP or LY294002. We demonstrated that VNS protected against burn injury–induced cardiac injury by attenuating mitochondria dysfunction, likely through the M_3_-AchR and the PI3K/Akt signalling pathways.

## Introduction

The pathogenesis of thermal injury is a complex process that is generated by the additive effects of inadequate tissue perfusion, free radical damage and systemic alterations in cytokine expression 1. Local burn insult leads to a systemic inflammatory response (SIRS) that is mediated by the release of pro-inflammatory cytokines such as tumour necrosis factor-a (TNF-a) and interleukin-1 (IL-1) 2, 3. Severe burn injury can also lead to cardiac stress by causing a surge of plasma catecholamines, which mediate the post-burn hypermetabolic response and result in an increase in sympathetic tone 4, 5.

The potent anti-inflammatory effect of efferent vagal nerve stimulation (VNS) plays a significant role in limiting the inflammatory response to injury 6. There has been significant focus on the signalling pathway by which VNS exerts its immunomodulatory effects, with specific attention paid to the alpha-7 subunit of the nicotinic acetylcholine receptor (α7nAChR) on macrophages 6. However, little information is available as to the protective effects offered by efferent VNS on cardiac injury induced by severe burn, and the role of cardiac muscarinic acetylcholine receptors (mAChR) in the protective effects of VNS.

Myocardial apoptosis is an important mechanism underlying burn-induced cardiac dysfunction and heart failure 7. There are two major apoptotic pathways: extrinsic (death receptor pathway) and intrinsic (mitochondrial pathway). In the intrinsic pathway, mitochondria play a central role in the integration and execution of a wide variety of apoptotic signals, including loss of growth factors, hypoxia, oxidative stress and DNA damage 7. Mitochondria provide the energy required for execution of apoptosis and the release of pro-apoptotic proteins such as cytochrome C and apoptosis-inducing factor (AIF) 7. VNS reportedly prevents myocardial reperfusion injury through inhibition of opening of mitochondrial permeability transition pore (mPTP) 8, suggesting that VNS may attenuate mitochondrial dysfunction and thereby inhibit myocardial apoptosis. In this study, we examined the deleterious effects of burn injury on heart tissue, and explored the effects of VNS on cardiac injury in a murine burn injury model, with a focus on the protective effect of VNS on mitochondrial dysfunction in the heart tissue.

## Materials and methods

### Animals

Male BALB/c mice (weighing 24–28 g) were purchased from Jackson Laboratories (Sacramento, CA, USA) and randomly assigned to nine groups (*n* = 5 per group): Sham, Burn (animals subject to burn injury only), 4-DAMP (animals subject to tail vein injection of 4-DAMP at 0.1 mg/kg only), LY (animals subject to tail vein injection of LY294002 at 1.5 mg/kg only), Burn/VNS (animals subject to burn injury and VNS right after burn), Burn/4-DAMP (animals subject to tail vein injection of 4-DAMP at 0.1 mg/kg for 15 min. before burn injury), Burn/VNS/4-DAMP (animals subject to tail vein injection of 4-DAMP at 0.1 mg/kg for 15 min. before burn injury and VNS), Burn/LY (animals subject to tail injection of LY294002 at 1.5 mg/kg for 15 min. before burn injury) and Burn/VNS/LY (animals subject to tail vein injection of LY294002 at 1.5 mg/kg for 15 min. before burn injury and VNS).

M_3_-mAchR antagonist 4-diphenylacetoxy-*N*-methylpiperidine methiodide (4-DAMP) was purchased from Santa Cruz Biotechnology, Inc (Santa Cruz, CA, USA) and phosphatidylinositol 3 Kinase (PI3K) inhibitor LY294002 was purchased from Cell Signaling (Danvers, MA, USA). This study was conducted in accordance with the institutional guidelines on the use of live animals for research, and the experimental protocol was approved by the Institutional Animal Care and Use Committee at University of California, San Diego.

### Burn injury

Animals were anaesthetized with inhaled isoflurane and their dorsal fur was removed with an electric clipper. Animals were placed in a template estimating 30% total body surface area and subjected to a thermal steam burn for 7 sec. as previously described 6, 9. Following burn injury, animals received a subcutaneous injection of 1.4 ml normal saline with 0.1 ml of buprenorphine (0.05 mg/kg) for pain control and pain resuscitation. At 6 and 24 hrs after burn, animals were again anaesthetized with inhaled isoflurane for tissue procurement.

### Vagal nerve stimulation

Immediately following burn, a right cervical neck incision was performed and the right cervical vagal nerve exposed. VNS was accomplished using a VariStim III probe (Medtronic Xomed, Jacksonville, FL, USA) with 2 mA at 1 Hz current, at 1 sec. intervals for 10 min. as previously described 6, 9. The incision was then closed with Vetbond (3M; St. Paul, MN, USA). Sham animals underwent right cervical incision and exposure of the vagal nerve, but did not receive stimulation.

### Histological evaluation

Heart tissue samples (*n* = 5 per group) were fixed in 10% neutral buffered formalin (Richard Allan Scientific, Pittsburgh, PA, USA). Sections (5 μm) were stained with Hematoxylin and Eosin by UCSD Histology Core Services. Myocardial histological score were evaluated by a pathologist blinded to the experimental groups. Three randomly selected fields from each specimen were graded according to the myocardial histological score system 10 with a scale from 0 to 2.0: 0 = no evidence of histologic injury; 0.5 = equivocal focal lesions; 1.0 = definite, but sparse lesions; 1.5 = less intense than 2, but more frequent than those graded a 1.0; 2.0 = florid and widespread lesions. Images were taken at 40× magnification using a light microscope (FSX100; Olympus, Center Valley, PA, USA).

### TUNEL assay

The paraffin sections were subject to TUNEL assay using the ApopTag InSitu apoptosis detection kit (S7111; Millipore, Billerica, MA, USA) according to the manufacturer's protocol. Cardiomyocyte apoptotic rates were calculated as the ratio of the numbers of TUNEL-positive cardiomyocytes to the number of total cardiomyocytes. Images were taken at 40× magnification with Fluorescence/Phase Contrast microscopy (FSX100; Olympus).

### Isolation of cytosolic and mitochondrial fractions

Cytosolic and mitochondrial fractions were isolated by using Pierce Mitochondria Isolation Kit for Tissue (Thermo, Pierce Biotechnology, Rockford, IL, USA) and according to the manufacturer's protocol. Briefly, heart tissue, harvested and snapped frozen and stored at −80°C previously, was pre-treated with 0.3 mg/ml trypsin and immersed in ice-cold reagent solution and homogenized on ice. Then the homogenate was centrifuged at 1000 × g for 10 min. at 4°C. The pellet was discarded and the supernatant was retrieved and centrifuged at 3000 × g for 15 min. at 4°C. The supernatant was retrieved again and centrifuged at 12,000 × g for 5 min. at 4°C. The resultant supernatant was designated as the cytosol. After adding wash buffer, the result pellet was again centrifuged at 12,000 × g for 5 min. at 4°C. The final pellet was designated as the mitochondrial pellet.

### Mitochondrial swelling assay

Fresh isolated mitochondria 0.8 mg/ml was incubated in 300 mM sucrose, 10 mM 3-[N-Morpholino] propanesulfonic Acid (MOPS; United State Biochemical Corporation, Cleveland, OH, USA) and Tris-HCl, pH7.4 for 15 min. at 26**°**C as previously described (9). After adding 800 μM Ca^2+^, mitochondrial swelling was monitored by the decrease in spectrophotometer absorbance at 540 nm in a multi-mode microplate reader (FLUOstar Omega; BMG Labtech).

### Determination of heart tissue ATP content

ATP content was determined by using ENLITEN® ATP Assay System Bioluminescence Detection Kit (FF2200; Promega, Madison, WI, USA) according to the manufacturer's protocol. Briefly, frozen heart tissues collected and stored as described above were homogenized in 10% trichloroacetic acid (TCA) and centrifuged at 5000 × g for 10 min. at 4°C. The supernatant was added to 50 mM tris-acetate, pH7.75 until the concentration of TCA was diluted to a final concentration of 0.1% and the ATP content was determined by a multi-mode microplate reader with luminescence luminometer (FLUOstar Omega; BMG Labtech).

### Western blot analysis

Protein samples of mitochondrial or cytosolic fractions were mixed with dithiothreitol (DTT) and SDS, boiled for 7 min. Equal amount of protein was loaded and subjected to 8–16% SDS-PAGE gels and immunoblotted by using the following antibodies: cytochrome C antibody (1:500; abcam, Cambridge, MA, USA), AIF antibody (1:1000; abcam), Bcl-2 antibody (1:100; abcam), phosphorylated Bad^136^ antibody (1:500; Cell Signaling, Danvers, MA, USA), Bad antibody (1:500; Cell Signaling), phosphorylated Akt^308^ antibody (1:1000; Cell Signaling), Akt antibody (1:1000; Cell Signaling), GAPDH antibody (1:1000; Cell Signaling) and ANT antibody (1:1000; abcam) respectively. The horseradish peroxidase-linked antirabbit IgG or horseradish peroxidase-linked antimouse IgG (1:2000; Cell Signaling) was used as the secondary antibody. The membrane was processed with Pierce Supersignal West Pico Chemiluminescent Kit and images were obtained with a Xenogen IVIS Lumina imagine system using the program Living Image 3.1 (Caliper Life Sciences, Hopkinton, MA, USA) and quantified by using UN-SCAN-IT gel Digitizing software (Silk Scientific, Orem, UT, USA).

### Statistical analysis

Statistical analysis was performed with SPSS 11.5 (SPSS, Chicago, IL, USA). Data values were expressed as means ± the standard error of the mean (SEM). The statistical significance among groups was determined by one-way analysis of variance (anova) with Bonferroni correction. Statistical significance was defined as *P* < 0.05.

## Results

### VNS attenuated burn-induced histologic heart injury

**Fig. 1:**
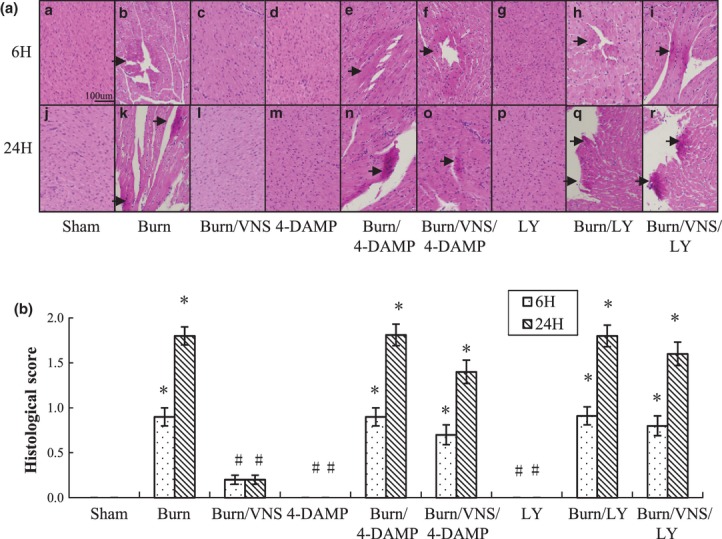
Histological analysis of heart tissue in experimental groups. Heart tissue sections (*n* = 5 per group) were stained with haematoxylin and eosin and viewed by light microscopy (**A)**. (a–i) Specimens obtained at 6 hrs after burn. (j–r) Specimens obtained at 24 hrs after burn. Black arrow shows early necrosis. Heart injury was scored on a scale of 0 (normal) to 2 (severe) by a pathologist blinded to the experimental groups (**B**). Images acquired at 40 × magnification. Black bar = 100 μm. *P < 0.01 *versus* Sham; #*P* < 0.01 *versus* Burn.

Burn caused significant cardiac pathological changes, including neutrophil infiltration, oedema, wavy fibres and coagulation necrosis (Fig. 1). VNS significantly reduced the pathological changes at both 6 and 24 hrs after burn, which the protective effects of VNS on cardiac injury were reversed in animals treated with the M_3_-mAchR antagonist 4-DAMP or PI3K inhibitor LY294002.

### VNS decreased cardiomyocyte apoptosis

**Fig. 2:**
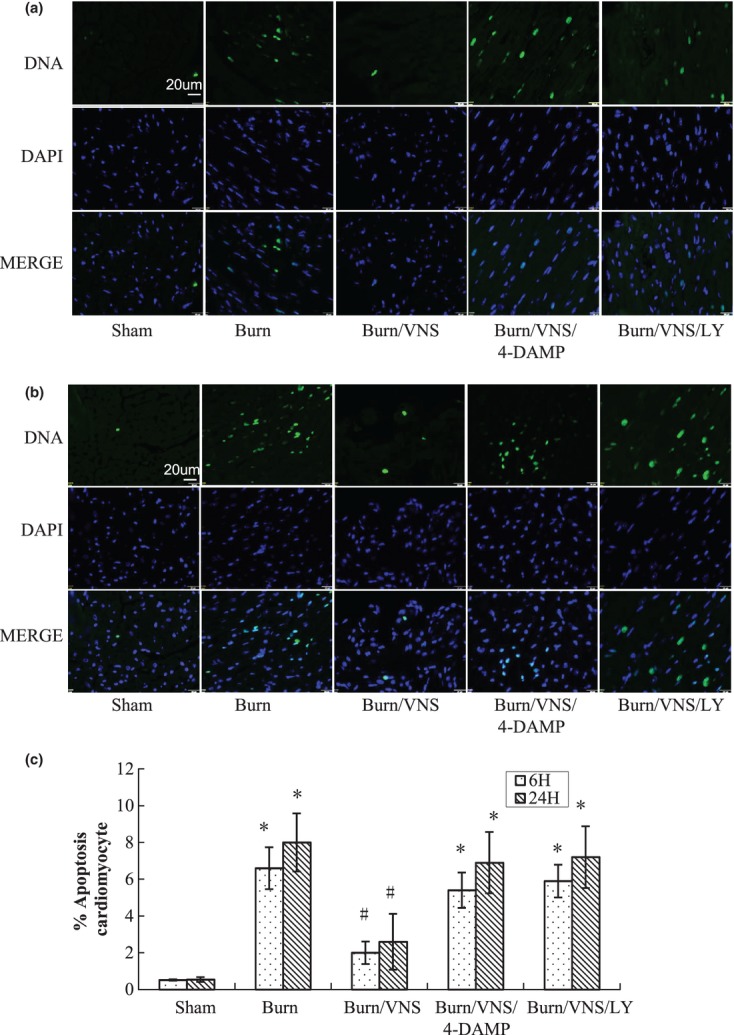
Cardiomyocyte apoptosis in experimental groups. Cardiomyocyte apoptosis rates (*n* = 5 per group) were determined with TUNEL staining 6 (**A**) and 24 hrs (**B**) after burn. Images demonstrated DNA fragmentation (green) and cell nuclei stained by 4′-6′ diamino-2-phenylindole (DAPI; blue). Apoptotic cardiomyocytes were shown in merge images. Cardiomyocyte apoptosis rate was calculated as the ratio of TUNEL-positive cardiomyocytes to the total cardiomyocytes (**C**). Images were acquired at 40× magnification. White bar = 20 μm. *P < 0.01 *versus* Sham; #*P* < 0.01 *versus* Burn.

Burn-induced cardiomyocyte apoptosis was observed at 6 hrs (Fig. 2A) and 24 hrs (Fig. 2B) after burn as measured by TUNEL staining. VNS significantly decreased the cardiomyocyte apoptotic rate. The protective effects of VNS on cardiomyocyte apoptosis were lost in animals pre-treated with 4-DAMP or LY294002.

### VNS inhibited burn-induced cardiac mitochondrial swelling and decrease in ATP content

**Fig. 3:**
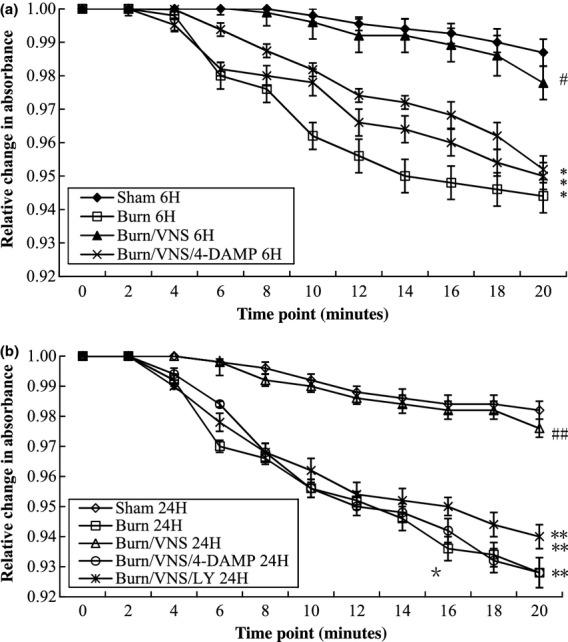
Mitochondrial swelling in experimental groups. Fresh mitochondria (*n* = 5 per group) isolated from heart tissue at 6 (**A**) and 24 hrs (**B**) after burn were subject to mitochondrial swelling assay. Mitochondrial swelling was monitored by the decrease in spectrophotometer absorbance at 540 nm. VNS inhibited the mitochondrial swelling that was observed after burn injury. The protective effect of VNS was inhibited by treatment with 4-DAMP or LY294002. *P < 0.05 *versus* Sham, **P < 0.01 *versus* Sham; #*P* < 0.05 *versus* Burn, ##*P* < 0.01 *versus* Burn.

**Fig. 4:**
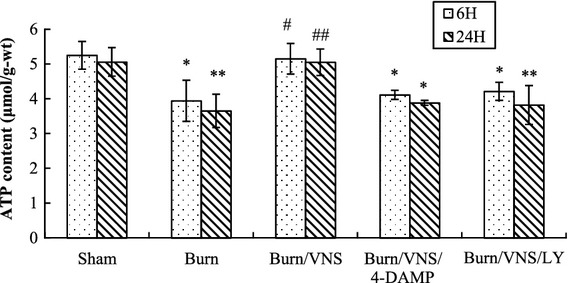
ATP Content in experimental groups. Fresh mitochondria (*n* = 4 per group) isolated from heart tissue at 6 (**A**) and 24 hrs (**B**) after burn underwent measurement of ATP content, which was determined by luminescence luminometer. VNS prevented the burn-induced increase in heart tissue ATP content. The protective effect of VNS was inhibited by treatment with 4-DAMP or LY294002. *P < 0.05 *versus* Sham, **P < 0.01 *versus* Sham; #*P* < 0.05 *versus* Burn, ##*P* < 0.01 *versus* Burn.

Compared with Sham, significant mitochondrial swelling was observed at 6 hrs (Fig. 3A) and 24 hrs (Fig. 3B) after burn. VNS effectively inhibited burn-induced mitochondrial swelling. Compared with Sham, the heart tissue ATP content decreased in the Burn group at 6 and 24 hrs after burn, which was inhibited by VNS (Fig. 4). The protective effects of VNS were inhibited by treatment with 4-DAMP or LY294002.

### VNS protected against burn-induced cardiac injury *via* Bcl-2 and the PI3K/Akt signalling pathway

**Fig. 5:**
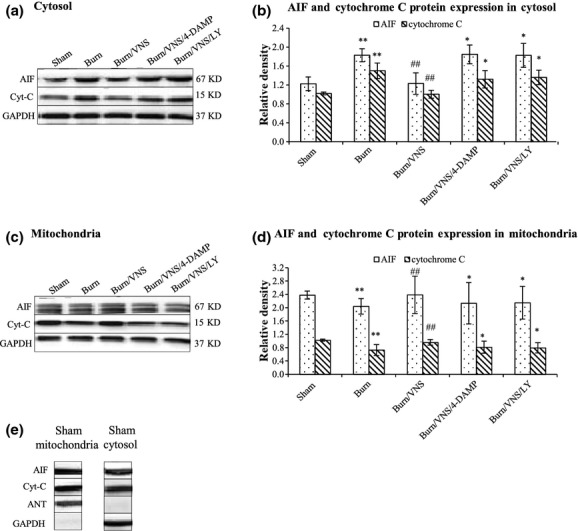
Western Blot Analysis of AIF and Cytochrome C Expression in Mitochondrial and Cytosolic Fractions of Heart Tissue. Cytosolic (**A**) and mitochondrial (**C**) fractions of heart tissue were isolated at 24 hrs after burn and underwent Western blot analysis for AIF and cytochrome C expression. ANT and GAPDH blotting were used as loading controls for mitochondrial and cytosolic proteins respectively. The density of the AIF and cytochrome C blot was normalized against that of GAPDH (**B**) and ANT (**D**) to obtain relative blot density. ANT was not detected in cytosolic fractions and GAPDH was not detected in mitochondrial fractions respectively (**E**). *P < 0.05 *versus* Sham, **P < 0.01 *versus* Sham; #*P* < 0.05 *versus* Burn, ##*P* < 0.01 *versus* Burn.

Apoptosis-inducing factor and cytochrome C protein expression in the cytosol were significantly increased at 6 (data not shown) and 24 hrs (Fig. 5A and B) after burn injury compared with Sham. This burn-induced increase in cytosolic AIF and cytochrome C was inhibited by VNS. AIF and cytochrome C expression was significantly decreased in the mitochondria at 6 (data not shown) and 24 hrs (Fig. 5C and D) and increased in the cytosol at 6 (data not shown) and 24 hrs after burn injury (Fig. 5A and B), which was inhibited by VNS. The cardiac-protective effects of VNS were lost in animals pre-treated with the inhibitors 4-DAMP or LY294002.

**Fig. 6:**
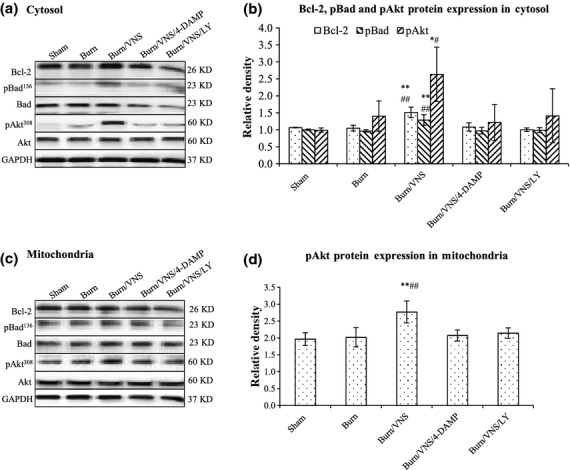
Western Blot Analysis of Bcl-2, Phosphorylated Bad (pBad^136^) and Phosphorylated Akt (pAkt^308^) Expression in Mitochondrial and Cytosolic Fractions of Heart Tissue. Cytosolic (**A**) and mitochondrial (**C**) fractions of heart tissue were isolated at 24 hrs after burn, and subjected to Western blot analysis for Bcl-2, pBad^136^/Bad and pAkt^308^/Akt expression. GAPDH and ANT were used as loading controls for cytosolic and mitochondrial proteins respectively. The density of the Bcl-2, pBad^136^ and pAkt^308^ blot was, respectively, normalized against that of GAPDH (**B**), and the density of the pAkt^308^ blot was, respectively, normalized against that of ANT (**D**) to obtain relative blot density. **P < 0.01 *versus* Sham; ##*P* < 0.01 *versus* Burn.

Bcl-2, phosphorylated Bad (pBad^136^) and phosphorylated Akt (pAkt^308^) protein levels were significantly increased in the cytosol by VNS at 6 (data not shown) and 24 hrs (Fig. 6A and B) after burn injury. The effects of VNS were reversed by treatment with 4-DAMP or LY294002 prior to burn injury. Mitochondrial levels of pAkt^308^ were significantly increased by VNS at 6 (data not shown) and 24 hrs (Fig. 6C and D) after injury. The effects of VNS were reversed by treatment with 4-DAMP or LY294002 prior to burn injury. Mitochondrial levels of both Bcl-2 and pBad^136^ were unchanged among the experimental groups (Fig. 6C).

## Discussion

In this study, we demonstrated that severe burn injury caused significant heart tissue pathological changes, cardiomyocyte apoptosis, mitochondrial swelling and decrease in heart tissue ATP content. These burn-induced changes were inhibited by VNS *via* attenuation of mitochondria dysfunction, likely through the M_3_-AchR and PI3K/Akt signalling pathways.

Mitochondria play a critical role in cardiomyocyte death *via* apoptosis and necrosis. Cellular dysfunctions induced by intra- or extracellular insults converge on mitochondria and induce mitochondrial dysfunction by the opening of mPTP, which leads to collapse of the membrane potential, mitochondrial swelling, ATP exhaust and release of pro-apoptotic proteins such as cytochrome C and AIF to activate energy-dependent apoptosis 11. Mitochondrial function can generally be predicted on the basis of cell ATP content 12. Mitochondrial swelling measures mitochondrial permeability transition, which may lead to myocardial apoptosis and necrosis. In addition, in response to apoptotic stimuli, pro-apoptotic Bcl-2 family members can induce mitochondrial crystal remodelling accompanied by outer membrane permeabilization and co-ordinated cytochrome C and AIF release from mitochondria, culminating in apoptosis 13. Thus, in this study, we measured heart tissue ATP content, mitochondrial swelling and mitochondrial and cytosolic level of cytochrome C and AIF to determine mitochondrial function. Our findings showed that burn injury caused mitochondrial dysfunction in heart tissue, which could be significantly attenuated by VNS.

The potent anti-inflammatory effects of efferent VNS have been shown to play a significant role in the prevention of the SIRS response to severe injury 14. Most previous studies on the ability of VNS to limit systemic inflammation have focused on the α7nAChR on macrophages 15. In this study, we explored the role of cardiac mAChR in the protective effects of VNS. The current view of cardiac mAchRs relates to the coexistence of multiple subtypes. More evidence is provided for the functional M_3_ receptors in mammalian hearts including mice 16. Functions of the cardiac M_3_-mAchR include regulation of heart rate, modulation of inotropic effects and cytoprotection against myocardial injuries 17–19. It has been reported that M_3_-mAchR activates anti-apoptotic signalling molecules 18. In our study, the M_3_-AchR-specific antagonist 4-DAMP 18, 20 prevented the cardioprotective effects of VNS, suggesting an important role of M_3_-AchR in VNS-induced protection from cardiac injury following burn. Further studies are needed to elucidate the role of M_3_-mAchR in the protective effects of VNS on the heart tissue.

There is abundant evidence that activation of the PI3K/Akt signalling pathway prevents cardiomyocyte apoptosis induced by multiple pathological insults, including ischaemia reperfusion, pressure overload, hypoxia, hypoglycaemia or cardiotoxic drugs 21, 22. The cardioprotective effects of Akt have been suggested to depend on its translocation from the cytosol to mitochondria where it inhibits opening of the permeability transition pore to maintain mitochondrial integrity 7, 23. Akt has several targets, including some of Bcl-2 family proteins, through which it may inhibit development of apoptosis. Bcl-2 family proteins play a central role in controlling mitochondrial apoptosis pathway. Akt prevents down-regulation of the anti-apoptotic protein Bcl-2 8 and phosphorylates Bad at Ser136, which result in inhibition of Bax/Bak-mediated pore formation, release of pro-apoptotic proteins, such as cytochrome C and AIF, and thereby inhibition of apoptosis 24. In our study, VNS significantly increased the phosphorylated Akt (pAkt^308^) both in mitochondria and cytosol, which was reversed by PI3K inhibitor LY294002. This suggested that VNS inhibited burn-induced cardiomyocyte apoptosis *via* the PI3K/Akt signalling pathway.

Bcl-2 is an important regulator of the mitochondrial apoptotic pathway. It has been shown to reduce the rate of ATP consumption during cardiac ischaemia by inhibiting the F_1_F_0_-ATPase, and to prevent permeability transition pore opening in heart mitochondria 25. A 3-step model for apoptotic cell death has been suggested: a pre-mitochondrial phase with activation of signal transduction cascades, a mitochondrial phase with permeabilization of the permeability transition pore and a post-mitochondrial phase with activation of apoptotic cascades by the proteins released from mitochondria to the cytosol. VNS induced the phosphorylated Bad, which were important factors on the pre-mitochondrial cell-survival cascades. The phosphorylation of Bad prevented its translocation to the mitochondria, thereby inhibiting its binding with Bcl-2, which was essential for maintaining the permeability transition pore in a closed state 8. In our study, VNS increased the level of Bcl-2 and phosphorylated Bad (pBad^136^) in cytosol. Although there is no significant change in mitochondria, these results still provided evidence for the ability of VNS to inhibit the three phases of apoptotic cell death. In addition, VNS significantly increased the Bcl-2 level in our study, which was in accord with our findings that VNS significantly decreased mitochondrial swelling and increased the heart tissue ATP content.

In summary, our study demonstrated that VNS attenuates burn-induced cardiac injury by attenuating mPTP opening and mitochondria dysfunction, likely through the M_3_-AchR and the PI3K/Akt signalling pathways. VNS may have clinical utility as a new therapeutic strategy aimed at improving cardiac function and increasing survival in patients with burn-induced cardiac dysfunction.
